# Supplementary material for: Cyclic Dichalcogenides Extend the Reach of Bioreductive Prodrugs to Harness Thiol/Disulfide Oxidoreductases: Applications to seco-Duocarmycins Targeting the Thioredoxin System
Source: ACS Cent Sci. 2023 Mar 28;9(4):763–76. doi: 10.1021/acscentsci.2c01465 (PMC10141580; doi:10.1021/acscentsci.2c01465)
Supplement: Supplementary file 3 — oc2c01465_si_003.pdf [file oc2c01465_si_003.pdf]

oc-2022-01465t.R1

Name: Peer Review Information for "Cyclic dichalcogenides extend the reach of bioreductive prodrugs to harness thiol/disulfide oxidoreductases: applications to *seco*-duocarmycins targeting the thioredoxin system"

First Round of Reviewer Comments

Reviewer: 1

Comments to the Author

The manuscript by Felber et al. reports the synthesis and development of a class of bioreductive cyclic dichalcogenide prodrugs to harness the thioredoxin system, an up-regulated process in cancer. The authors demonstrate an interesting strategy for specific bioreduction-based release to activate duocarmycin prodrugs. The work is presented well and the data is thoroughly described in the manuscript. The body of work and data collected is impressive and spans organic synthesis, in vitro and in cellulo experiments, as well as in vivo assays in mouse models, a body of work that all contributes to support the goals and hypotheses of the study. I congratulate the authors on their achievements.

Overall the work is appropriate for publication in ACS Central Science, however I have two concerns regarding the selectivity and mechanism of uptake of the system that might be addressed prior to publication.

(1) The authors indirectly demonstrate selectivity for the dichalogenide prodrugs with thioredoxin by comparing the resistance to disulfide reduction in the presence of GSH to the significant unmasking of the active pharmacophore with TCEP. Based on this information as well as previous insight from prior work by the authors it is inferred that this new system will be both monothiol resistant as well as Trx-activatable. However, the direct activation of the duocarmycin prodrugs by Trx is not shown and cell-based assays are employed instead. The cell-assays show that Trx plays a role in the activation of the prodrug, but do not present a clear picture regarding the actual mechanism at play. As it is stated within the manuscript that other thiols and cellular reductases may be acting upon the system, Trx-selectivity is something which needs to be better addressed in some fashion—such as through a Trx-based in vitro assay—in order to support the claims of the authors.

(2) It is stated that “Testing whether the cellular activation of the prodrugs is due to their intended target, thioredoxin, is a non-trivial task: since there are no stable Trx knockouts, nor are pharmacologically clean cellular Trx inhibitors available.” In the cellular assays a negative control is applied with a non-reducible carbamate analogue, but no information is presented regarding off-target (non-Trx) activation of the prodrug and no information regarding correlating Trx expression levels with cell-specific activity is shown. Given the relatively modest potencies produced, in addition to the potential complexity of cellular uptake, it seems possible that some degree of non-selective activation is occurring. Some comment or investigation in this matter would provide useful insight to the reader

regarding the intricacies of the system. For example, is there a second reductase involved or is some mechanism of thiol-mediated cellular uptake and cellular entry occurring (similar to what has been developed by Matile and co-workers, albeit with 5-membered chalcogenides), a process which could also theoretically activate the prodrug during cell uptake and cause the results observed? If involved, inhibition of this process could improve the potency of the approach (Cheng et al. Chem. Sci. 2021, 12, 626– 631).

One additional comment:

Figure 1a: “Nitroaromats” should be changed to the English nomenclature.

Reviewer: 2

Comments to the Author

This good paper demonstrates the possibility of prodrug release through reductive activation with the reducing enzyme thioredoxin. While the quality of chemistry and cellular experiments is excellent, I think there are some key missing points before the paper should be published.

1. Have the TrxR1 knockouts been validated? Where are they from?

2. The reductive activation levels in MEF cells are moderate (2-4 fold), it is unlikely this would drive selective efficacy (consistent with lack of efficacy in most of the mouse studies)

3. A dose-response would have been convincing. For example, in the MEF knockouts, what happens if TrxR1 is rescued by ectopic expression? Is toxicity increased?

4. The mouse studies are rather confusing and difficult to conclude anything from in my view. The compounds seem tolerated, but efficacy is not observed in most cases. Only in the animals with no control compounds was there a sign of efficacy, without the control, though, I don't see how anything can be concluded there.

5. In general I would argue that using end-point toxicity or viability assays to quantitate the effect is rather dangerous as the only method, since such assays have large variations.

6. A better rationale (showing cancer data with overactive reduction as a phenotype, for example) for why reductive activation is a plausible approach would have been welcome.

Author's Response to Peer Review Comments:

All requests and comments have been addressed; see the attached Replies file.

ACS Central Science
Prof Editor

Dr. Oliver Thorn-Seshold
Faculty of Chemistry and Pharmacy
Ludwig-Maximilians University, Munich
5-13 Butenandtstr, Munich 81377, Germany
oliver.thorn-seshold@cup.lmu.de; +49 89 2180 77246
22 February 2023

Dear Reviewers, Dear Prof. Editor,

We are very grateful for the straightforward and positive review process and we look forward to the opportunity to publish our revised manuscript ***Cyclic dichalcogenides extend the reach of bioreductive prodrugs to harness thiol/disulfide oxidoreductases: applications to seco-duocarmycins targeting the thioredoxin system*** in ACS Central Science.

We want to highlight how thankful we are for your reviewing time and investment - this was an excellent level of review and we were delighted with the positive outcome.

We responded to all reviewer comments and implemented all formatting changes, as below:

Reviewer #1:

Recommendation: *Publish in ACS Central Science after minor revisions noted.*

Comments: *The manuscript by Felber et al. reports the synthesis and development of a class of bioreductive cyclic dichalcogenide prodrugs to harness the thioredoxin system, an up-regulated process in cancer. The authors demonstrate an interesting strategy for specific bioreduction-based release to activate duocarmycin prodrugs. The work is presented well and the data is thoroughly described in the manuscript. The body of work and data collected is impressive and spans organic synthesis, in vitro and in cellulo experiments, as well as in vivo assays in mouse models, a body of work that all contributes to support the goals and hypotheses of the study. I congratulate the authors on their achievements.*

- We thank the reviewer for this strong and positive assessment.

Overall the work is appropriate for publication in ACS Central Science, however I have two concerns regarding the selectivity and mechanism of uptake of the system that might be addressed prior to publication.

(1) The authors indirectly demonstrate selectivity for the dichalogenide prodrugs with thioredoxin by comparing the resistance to disulfide reduction in the presence of GSH to the significant unmasking of the active pharmacophore with TCEP. Based on this information as well as previous insight from prior work by the authors it is inferred that this new system will be both monothiol resistant as well as Trx-activatable. However, the direct activation of the duocarmycin prodrugs by Trx is not shown and cell-based assays are employed instead. The cell-assays show that Trx plays a role in the activation of the prodrug, but do not present a clear picture regarding the actual mechanism at play. As it is stated within the manuscript that other thiols and cellular reductases may be acting upon the system, Trx-selectivity is something which needs to be better addressed in some fashion—such as through a Trx-based in vitro assay—in order to support the claims of the authors.

- Thanks for this insightful observation. **The simple response** which we have now done, is testing for direct prodrug activation by purified Trx *in vitro* as suggested by the reviewer, with readout of speciation over time being acquired by HPLC-DAD-MS (**new Fig S7**; experimental details in the Supp Info at that location; figure reproduced below). This assay confirms that e.g. both Trx and TrxR are capable of activating SS66C-type prodrugs; whereas SeS60-type prodrugs are directly activated by TrxR with little extrra

contribution from Trx. Both results matched the expectations we had given in the text, as based on our previous work on cyclic dichalcogenides as we cited (see also section 2.4). => thus, this assay does support the role of the Trx system in prodrug activation.

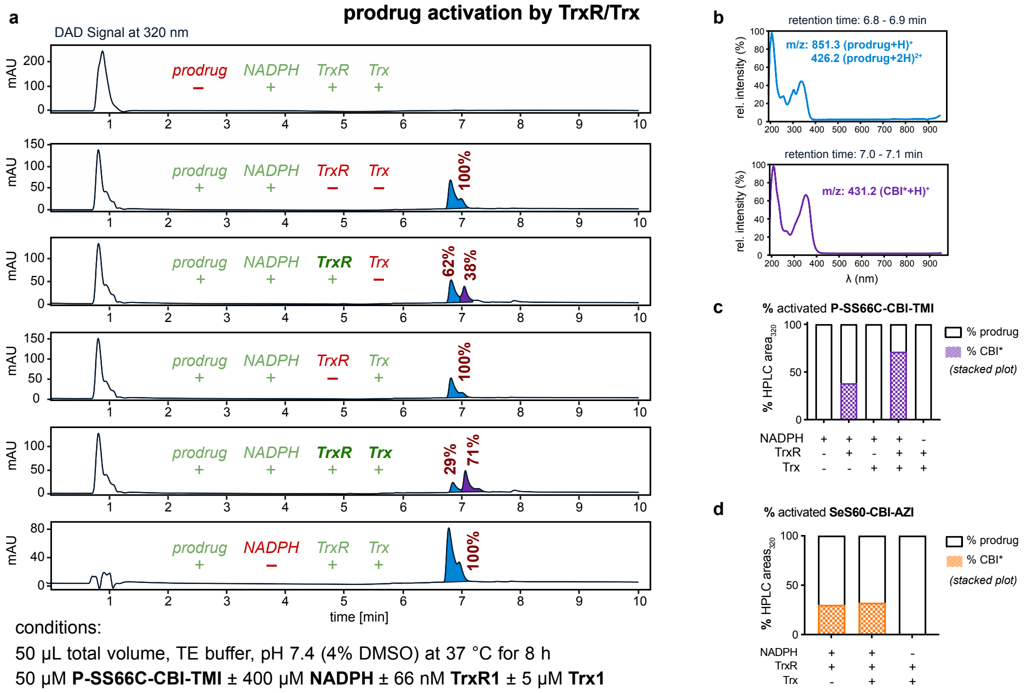

(2) It is stated that "Testing whether the cellular activation of the prodrugs is due to their intended target, thioredoxin, is a non-trivial task: since there are no stable Trx knockouts, nor are pharmacologically clean cellular Trx inhibitors available." In the cellular assays a negative control is applied with a non-reducible carbamate analogue, but no information is presented regarding off-target (non-Trx) activation of the prodrug and no information regarding correlating Trx expression levels with cell-specific activity is shown. Given the relatively modest potencies produced, in addition to the potential complexity of cellular uptake, it seems possible that some degree of non-selective activation is occurring. Some comment or investigation in this matter would provide useful insight to the reader regarding the intricacies of the system. For example, is there a second reductase involved or is some mechanism of thiol-mediated cellular uptake and cellular entry occurring (similar to what has been developed by Matile and co-workers, albeit with 5-membered chalcogenides), a process which could also theoretically activate the prodrug during cell uptake and cause the results observed? If involved, inhibition of this process could improve the potency of the approach (Cheng et al. Chem. Sci. 2021, 12, 626– 631).

- We completely agree. Reductive activation by enzyme X in a cell-free system only indicates the same is *possible* in cells: there is no proof that it actually *does* happen, nor does it suggest that it is the only route for reductive activation. We have addressed these issues just after Fig 9 in the main text, and in Supporting Section 6.3.
  Regarding ***"off-target (non-Trx) activation of the prodrug,"*** we argue here and in the previous papers we reference, that the true cellular activity of a reductase on a probe is controlled by the enzyme's compartmentalisation, PTMs / redox poise, and by preferential binding to its native (competitive) substrates instead of to our prodrugs (more discussion in e.g. doi.org/10.1016/j.chempr.2022.03.010). This is why we are cautious to say only that e.g. our cellular TrxR1 knockout data indicates the role of "the Trx system" rather than e.g. Trx1 as a defined cellular actor. (By contrast, a reaction that does *not* happen *in vitro*, and where the biological reductant does not have concentration / localisation-based activity control, is *not* likely to happen in cells. This is the case e.g. for the non-reactivity of the SS66C-type prodrugs with monothiols: which is why we are confident of their monothiol/GSH stability in cells.)
  To understand cellular activation profiles properly requires a comprehensive battery of

knockout / knock-back / inhibition studies. However, that is such a large and specialised undertaking that it is outside the scope of this paper; we have instead built it into a different "profiling" paper that we recently preprinted (doi.org/10.26434/chemrxiv-2023-tm21m), which over 8 main figures and 150 pages of SI performs just such a battery of selectivity tests on the cyclic dichalcogenide redox substrate motifs we use in this paper (reference 19 in the revised paper). As we note on page 3 now, *"a more complete view of the cellular selectivity profiles of these dichalcogenides is given in reference 19."*

*[Of course the reviewer is right - there are certainly nonspecific activation mechanisms also at work. **Here, for interest, are our speculations** what they are: (A) In our opinion, the most likely other reductase that can be involved in reductive activation is Grx2, as we have seen in the profiling paper (its Fig 7 and related supporting information); however, we also note that the profiling paper indicates through three orthogonal assays that the selenium-dependent TrxR system is (indirectly or directly) responsible for half or more of the cellular activation of probes like SS66C, i.e. "SS66C is primarily a reporter on Trx system activity" is correct. (B) We believe the most significant nonreductive mechanism is the baseline of non-redox prodrug activation that is occurring with our secondary carbamate designs, as shown by the non-reducible control prodrugs **CC60** and **O56** which we designed and included to test just this possibility. Such activation is not surprising, and may stem from e.g. inefficient hydrolytic processing of the carbamate by carboxylesterases, which we reference (a classic example is the duocarmycin piperazinyl carbamate prodrug Pibrozelesin). In this paper our data suggests that nonreductive activation is responsible for ca. 16-30% of the total activation of SS66C (nonreducible vs reducible potencies). (A+B) Taken together, those already account for >66 - >80% of cellular activation. (C) We do not think that potential activation during thiol-mediated uptake can be significant, because the nonpolar environment should block the irrreversible 5-exo-trig step; however, we have recently shipped most of these redox substrates to Matile to examine how significant TMU can be in the overall cellular entry/enrichment of such prodrugs.]*

- Regarding ***"correlating Trx expression levels with cell-specific activity"***, we provided Fig S14 / Supporting Section 6.3 (Test for correlation of oxidoreductase proteomic expression levels, to potency or reductive index) as well as the note at Table S2 (correlation to mRNA levels) which address this. The problem is that true *activity* is unknown; only some data on 'expression' (usually PCR data) are available, which is a very bad proxy for S/Se reductase activity (e.g. TrxR, like other selenoproteins, has wildly different protein levels from mRNA levels; and protein levels also do not correlate with activity, due to various post-translational regulations). More discussion at Review 2.
- We believe that the combination of the HPLC assay, the citations to the comprehensive profiling paper, and Supporting Section 6.3, address the reviewer's two points to the fullest extent possible in the current paper. We too look forward to future research that can uncover quantitative detail for redox-prodrug activation routes, but we think that this paper provides large enough advances beyond the state of the art to be satisfactory.

One additional comment:

Figure 1a: “Nitroaromats” should be changed to the English nomenclature.

- Done.

***Additional Questions**:*

Quality of experimental data, technical rigor: Top 5%

Significance to chemistry researchers in this and related fields: Top 5%

Broad interest to other researchers: High

Novelty: High

Is this research study suitable for media coverage or a First Reactions?: No

- In summary, we thank the Reviewer 1 for their time, expertise, and close attention, and we consider that the new Fig S7 plus the separate preprint addresses their comments.

Reviewer #2:

Recommendation: *Reconsider after major revisions noted.*

Comments:

This good paper demonstrates the possibility of prodrug release through reductive activation with the reducing enzyme thioredoxin. While the quality of chemistry and cellular experiments is excellent, I think there are some key missing points before the paper should be published.

1. Have the TrxR1 knockouts been validated? Where are they from?

- This is cited as reference 48. They have been generated and published around 2008-2010 by Marcus Conrad, and distributed and validated widely in the community since. We now added this explicit information in the Supp Info in the Cell Culture section.

2. The reductive activation levels in MEF cells are moderate (2-4 fold), it is unlikely this would drive selective efficacy (consistent with lack of efficacy in most of the mouse studies)

- We think the situation is different; also, our mouse studies do show selective efficacy for the reducible prodrugs (see e.g. Fig 10d and point below).
  The "2-4 fold" refers to the fold-changes of potency upon knockout of a single isoform of a single reductase in a non-cancer MEF cell line (Fig 7). MEF cells are not the intended target of the prodrugs - they are a reporter system where a validated TrxR1 knockout is available that can directly showcase if a significant fraction of drug activation is due just to Trx1 system; "4-fold enhancement" can also be expressed as saying "in total, a maximum of 25% of prodrug activation occurs by all mechanisms taken together *except* the 75% direct activation by TrxR1/Trx1" which is actually exceptional for a first report. We would also direct attention to Fig 8, where some cancer cell lines show up to 30-fold higher reductive activation (by all reductases combined) than non-reductive, i.e. 97% reductive pathways. These aspects are clarified in the main text in sections 2.6-2.7.
  However, there is a large additional gap separating the knowledge that the major *routes* of activation are reductive, from the hope to observe *selective anticancer efficacy* in tumour indications. Indeed, the field of C- and N-based bioreductive prodrugs has hitherto failed to jump this gap, despite decades and billions spent. It was our hypothesis, and one that our data do not contradict, that by harnessing a novel S/Se-based chemical reactivity manifold for bioreductive activation (see the up to 97% thiol/disulfide-based reductive activation indices plotted over 176 cell lines in Fig 8), we introduce a new scope for bioreductive drugs, and a new chance for them to clear this gap. From these promising initial results, it will be interesting to see what progress further years and funding will enable. This is clarified in the Introduction and in the Conclusions.

3. A dose-response would have been convincing. For example, in the MEF knockouts, what happens if TrxR1 is rescued by ectopic expression? Is toxicity increased?

- We did not perform that type of rescue test, but the difference between the parental Cre line and the excised KO line (Fig 7) is strongly indicative what it would show. All the same, we would like to remind that MEF cells and even TrxR1 are not the targets of interest: the point of the study is to demonstrate thiol/disulfide manifold dependency, which is done over the other 176 cell lines. As the reviewer also notes in point 5, short-term noninvasive reporter assays are better gauges of dose-response than long-term toxicity; and we have addressed that in the new preprint we now cite (reference 19).

4. The mouse studies are rather confusing and difficult to conclude anything from in my view. The compounds seem tolerated, but efficacy is not observed in most cases. Only in the animals with no control compounds was there a sign of efficacy, without the control, though, I don't see how anything can be concluded there.

- We argue that the 4T1 assay (Fig 10b, 10d) which included the non-reducible control actually does show a clear efficacy difference between poor to moderate efficacy hydrolytic P-CC60, and the strongly effective P-SS60 series (same efficacy as benchmark clinical therapeutic). Smaller tumours are typically more sensitive to trace amounts of chemotherapeutics, therefore it is unsurprising that the large differences in efficacy become apparent as the tumour size increases over time (endpoint: Fig 10d). Thanks to this comment, we changed the graphical representation in Fig 10b in a way that we think highlights that the control set was run in 4T1, and we consider that this addresses the referee's comment.

  *[**For interest**: we too would have liked to run several more groups in parallel to cover non-reducible controls also in the BxPC-3 model, and we would have liked to cover many more families of prodrug (e.g. SS66C-type), and we would have liked to test many more tumour models not just 4T1 and BxPC-3. Such extended assays would have provided a more comprehensive view over efficacy differences and the role of bioreduction. However, (a) such studies are very costly and time-consuming, and our funding is now used up; and (b) we do not think that simply giving more assays would deliver conceptually new information with a sufficient cost-benefit ratio. The next really important question to be tackled is, as the reviewer highlights in point 5, "how much reductive and how much non-reductive activation are actually occurring in different tissue types in vivo". That requires a completely different set of compounds that are noninvasive acute reporters (see reply to point 3), and it is the topic of a separate major paper we are working on at the moment. So, we are content that the vast majority of the scientific challenges in this large paper have been addressed over the first 9 figures of design, synthesis, characterisation, biochemistry, cellular reductive validation, 177-cell-line profiling, and in vivo tolerability; and we consider that a sufficiently encouraging level of reduction-dependent in vivo efficacy has been provided in Fig 10; thus we consider that the proper scope of the paper is covered.]*

5. In general I would argue that using end-point toxicity or viability assays to quantitate the effect is rather dangerous as the only method, since such assays have large variations.

- Completely true. We could not agree more. That is why we had written in the Conclusions, *"the ideal goal for redox prodrugs is to develop a platform approach that can maximise the ratio of drug exposure in tumoral vs. in healthy tissues, rather than relying only on differences of their intrinsic sensitivities to a given cargo. **Testing this exposure ratio, with directly quantifiable redox reporter prodrugs based around another cargo than the CBIs, is our aim in ongoing work.** Quantifying exposure with reporters, and developing increasingly effective prodrug-based therapeutics, are mutually reinforcing advances for testing the potential of bioreductive prodrugs. We believe that both will be required, over multiple interleaved cycles of refinement, in order to face this multi-variable problem with a quantitative, SAR-based understanding...".* We think this highlights our and the reviewer's shared opinion, and tells the reader what our next steps are to address it, so we consider that the manuscript covers this issue well.

6. A better rationale (showing cancer data with overactive reduction as a phenotype, for example) for why reductive activation is a plausible approach would have been welcome.

- First, in the main text we do direct the reader to our potency vs reductase proteomic expression level correlation tests over the ~60 cell line panel (Supporting Section 6.3, Fig S14) which were performed for this goal, using the available data; we do also mention gene transcript correlation testing (*"mRNA transcript analysis ...(see **Table S2**)."*)
  Second, in a broad sense, true activity (turnover) data has been reported for decades as the basis for all investment into the field of bioreductive prodrugs (C-, N-, and other chemotypes), and we give citations to it in the Introduction. The reasonable assumption is that excess bioreductive activity in one chemical manifold probably indicates similar excesses in others: but the problem facing our field is that for S/Se-based manifolds, *there is simply no appropriate S/Se-activity-data available, because until now there have been no selective chemotypes developed that could report on them* (see reply to the 2nd comment of Reviewer 1). That is the background against which our chemistry represents

a novel and urgently needed step. There are multitudes of studies that talk about changes of mRNA transcript levels coding for selected dithiol oxidoreductases because that is cheap and easy to measure. There are far fewer studies truly assessing some of their protein expression levels, because that is much more expensive to do quantitatively (e.g. Ab-based methods). But there are no reliable reports on S/Se-reductase activity profiling, because there have been no methods to detect or harness it - so there is no literature to fall back on. We are just as frustrated as the referee by this situation; but we are working hard to apply our reporter variant cyclic dichalcogenide probes to start performing such activity analysis across physiological and pathological conditions (not just cancers), and with some luck, the next 5-10 years will start to see hard data accumulating, as we write in the Conclusions.
We thus consider that this point is clearly raised for the reader, is addressed in Section 6.3 to the best of our current abilities, and that the next steps which we believe will crack the problem are laid out transparently.

Additional Questions:

Quality of experimental data, technical rigor: High

Significance to chemistry researchers in this and related fields: High

Broad interest to other researchers: High

Novelty: High

Is this research study suitable for media coverage or a First Reactions (a News & Views piece in the journal)?: No

- We thank the Reviewer 2 for their time, expertise, and close attention, and we consider that updated Fig 10, referencing, & cell line detail, satisfactorily address their comments.

Editorial / Formatting changes requested:

TITLE: Please ensure that the title matches exactly on the manuscript, Supporting Information file, and in Paragon Plus.

- Done.

AFFILIATIONS: Please add a country to the affiliation statement in your manuscript and Supporting Information file.

- Done.

ABSTRACT: Please shorten the abstract to 200 words or less.

- Done.

FIG. 3: Please ensure Figure 3's caption appears directly below it, not on a separate line.

- Done.

SI STATEMENT: A brief description of the supplementary material in the manuscript.

- Done.

TOC GRAPHIC: ACS Central Science requests a Table of Contents (TOC) Graphic.

- Done.

SYNOPSIS: ACS Central Science requires a brief synopsis.

- Done.

Other changes:

- no major changes of content or message, no significant reference additions

- we acquired additional data for the potencies of the CBI-AZI set of compounds across nearly 60 cell lines. Their potencies and reductive indices are given in **New Table S3b**, which provides an even more comprehensive view of the ***trigger-dependency*** (and cargo-independency) of the reductive activation index - one of the most important results of our paper.

- Thanks to that extra data, we were also now able to do several pairwise comparisons across ~60-cell-line potency data for (i) prodrugs differing only in cis vs trans stereochemistry; (ii) linear vs cyclic disulfide topologies; (iii) TMI vs AZI cargos with otherwise identical redox triggers; and those comparisons are shown in **New Figure S13**.

- Finally, we provide a new supporting Excel spreadsheet file **Data S1** that has conveniently formatted HTS potencies and reductive indices for all ten prodrugs across all 176 HTS cell lines, as well as the source data for the protein expression/activity correlation test. We hope that this new datafile will allow researchers to test their own hypotheses about reductive activation (see point 6 from reviewer 2), and that as new acute reporter data hopefully become available over the next years, this datafile will provide a lasting useful record of apparent reductive indices in long-term assays that can be repeatedly harvested and analysed as a predictive basis for employing this set of redox triggers to gate the reporter or drug activity of other cargos.

We therefore consider all comments and requests have been addressed. We also believe that we have proactively increased the comprehensiveness and the accessibility of the datasets underlying the research in this manuscript.

We thus consider our manuscript ready for publication and we look forward to your feedback.

With best regards, and thanks for your time and efforts -

Dr. Oliver Thorn-Seshold, on behalf of all coauthors

oc-2022-01465t.R2

Name: Peer Review Information for "Cyclic dichalcogenides extend the reach of bioreductive prodrugs to harness thiol/disulfide oxidoreductases: applications to *seco*-duocarmycins targeting the thioredoxin system"

Second Round of Reviewer Comments

Reviewer: 2

Comments to the Author

the authors have addressed the voiced concerns adequately. There is only one additional point I would like make which is listed as point 5 from reviewer 2: The author answers as if this is the next step in the process. In general for key conclusions in biology at least two independent key dataset types are needed to present a compelling picture. To me this paper still relies too much on endpoint toxicity data for its key conclusions.

Reviewer: 1

Comments to the Author

The revised manuscript by Felber et al. addresses the previously stated concerns to a satisfactory level such that I feel it is ready for publication.

Author's Response to Peer Review Comments:

Dear ACS Central Science, Dear Prof Editor, Dear Reviewers,

Thank you so much for this excellent news. All changes done:

(1) AU EMAIL: Please label as "email." => done

(2) CAPTION PERMISSIONS => I confirm that all references in the captions pertain to chemical structures used, not to the image itself.n No images have been previously published.

(3) TOC PAGE: => OK, removed title and author list

and a note for original Reviewer 2 in the cover letter.

We look forward to publishing in ACS Central Science!

All the best,

Oliver on behalf of all coauthors
